# Hereditary neuropathy with liability to pressure palsy (HNPP): report of a family with a new point mutation in *PMP22* gene

**DOI:** 10.1186/s13052-017-0414-4

**Published:** 2017-10-27

**Authors:** Carlo Fusco, Carlotta Spagnoli, Grazia Gabriella Salerno, Elena Pavlidis, Daniele Frattini, Francesco Pisani

**Affiliations:** 1 Department of Pediatrics, Child Neurology Unit, Santa Maria Nuova Hospital, IRCCS, viale Risorgimento 80, 42123 Reggio Emilia, Italy; 2 Department of Pediatrics, Pediatric Neurophysiology Laboratory, Santa Maria Nuova Hospital, IRCCS, viale Risorgimento 80, 42123 Reggio Emilia, Italy; 3 Child Neuropsychiatry Unit, Medicine & Surgery Department, University of Parma, via Gramsci, 14, 43126 Parma, Italy

**Keywords:** HNPP, PMP22, Neuropathy, Childhood, Point mutation

## Abstract

**Background:**

Hereditary neuropathy with liability to pressure palsy (HNPP) is an autosomal dominant disorder most commonly presenting with acute-onset, non-painful focal sensory and motor mononeuropathy. Approximately 80% of patients carry a 1.5 Mb deletion of chromosome 17p11.2 involving the peripheral myelin protein 22 gene (PMP22), the same duplicated in Charcot-Marie-Tooth 1A patients. In a small proportion of patients the disease is caused by *PMP22* point mutations.

**Case presentation:**

We report on a familial case harbouring a new point mutation in the *PMP22* gene.

The proband is a 4-years-old girl with acute onset of focal numbness and weakness in her right hand. Electroneurography demonstrated transient sensory and motor radial nerves involvement. In her father, reporting chronic symptoms (cramps and exercise-induced myalgia), we uncovered mild atrophy and areflexia on clinical examination and a mixed (predominantly demyelinating) polyneuropathy with sensory-motor involvement on electrophysiological study.

Both carried a nucleotidic substitution c.178 + 2 T > C on intron 3 of the *PMP22* gene, involving the splicing donor site, not reported on databases but predicted to be likely pathogenic.

**Conclusions:**

We described a previously unreported point mutation in *PMP22* gene, which led to the development of a HNPP phenotype in a child and her father. In children evaluated for a sensory and motor transient episode, HNPP disorder due to *PMP22* mutations should be suspected. Clinical and electrophysiological studies should be extended to all family members even in the absence of previous episodes suggestive for HNPP.

## Background

Peripheral neuropathies in childhood differ from those of adult-onset, because in most cases they are genetically-determined (versus acquired) and show considerable phenotypic and molecular genetics heterogeneity. Based on adult prevalence studies, Charcot-Marie-Tooth (CMT) is the commonest neuromuscular disorder, typically presenting with distal wasting and weakness, decreased deep tendon reflexes, and frequently contractures and skeletal deformities [1]. However, additional clinical signs, such as marked sensory involvement, respiratory compromise, upper limb involvement, visual or hearing impairment, pyramidal signs and intellectual disability can be present, implying a further need to widen diagnostic investigations, to include complicated forms of hereditary spastic paraplegia, or inborn errors of metabolism or neurodegenerative disorders [2–4]. Furthermore, the coexistence of central and peripheral nervous system involvement does not rule out hereditary peripheral neuropathy [5].

Hereditary neuropathy with liability to pressure palsy (HNPP) is an autosomal dominant disorder first described by De Jong in 1947. The most common presenting symptom is the acute onset of a non-painful focal sensory and motor mononeuropathy [6]. The clinical picture consists of recurrent focal neuropathies at entrapment sites or susceptible pressure points [7], presenting with either peroneal or ulnar neuropathy in 70% of cases, and usually preceded by a trivial trauma, minor compression or physical exercise [6]. Rarely, HNPP can present with atypical symptoms including progressive mononeuropathies, chronic sensory neuropathy, transient sensory symptoms or Charcot-Marie-Tooth-like presentation [8].

Approximately 80% of patients carry a 1.5 Mb deletion of chromosome 17p11.2 involving the peripheral myelin protein 22 gene (*PMP22*). In a small number of patients the disease is caused by *PMP22* point mutations [8]. The same gene leads to CMT 1A when duplicated. CMT 1A is an autosomal dominant demyelinating polyneuropathy usually presenting in the first decade of life with progressive weakness and wasting of the extensor muscles of the feet and toes, and later involvement of the distal upper limb muscles. Congenital cases have also been described, exceptionally with pes cavus as an isolated finding [9].

The proposed pathophysiological mechanism for the development of a HNPP phenotype is the under-expression of the *PMP22* gene, either by deletion of one gene copy or by inactivation of one gene copy by mutation. PMP22 is a transmembrane protein located in the compact myelin of all myelinated fibers of the peripheral nervous system. Its deficiency disrupts myelin junctions resulting in impaired propagation of nerve action potentials [10]. In this article we report on clinical and neurophysiologic findings of a 4 years-old patient and her father carrying a new point mutation in the *PMP22* gene and with clinical symptoms fulfilling the diagnostic criteria for HNPP.

## Case presentation

**Fig. 1:**
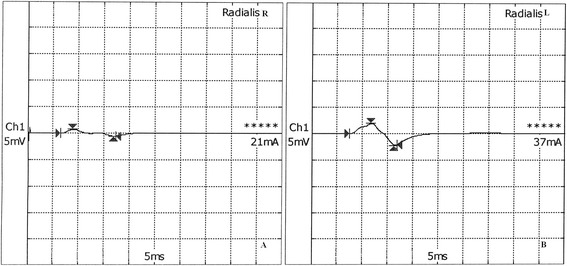
Motor conduction velocity in the right (**a**) and left (**b**) radialis nerves of our proband. Reduction of motor radial compound muscle action potential is evident in the right hand compared with contralateral radial nerve examination (1.4 mV versus 3.2 mV)

The proband was a 4 years-old previously healthy girl who complained of acute onset prolonged right hand numbness in the dorsal surface of the lateral three and half digits, and their associated palm area (superficial branch of radial nerve), following minor compression (she had been leaning on her hand after falling asleep on a bus). She was born from non-consanguineous parents with a normal birth. Upon history taking, all early developmental milestones were reported as normally achieved. The neurological examination revealed mild hypostenia in extensor digitorum communis and indicis right muscles and hypoesthaesia in the hand area innervated by radial nerve. No pes cavus or planus, wasting and weakness of foot muscles and of peroneal and anterior tibialis muscles or limb areflexia were noticed. The first electrophysiological examination showed a symmetrical mild reduced sensory and motor conduction velocity with normal distal latencies and mild reduction of compound action muscle potential amplitude. Moreover, a reduction of motor radial muscle action potential was demonstrated in the right hand compared with contralateral radial nerve examination (Fig. 1). Laboratory tests, including routine hematology, renal function, electrolytes, lactate, and urinalysis, were all within normal limits. The apolipoprotein and lipid profile, alpha-fetoprotein, lactate, vitamin E, vitamin B12 anti-ganglioside antibodies and serum-free thyroid hormone measurements were all normal. Molecular genetic analysis ruled out duplications or deletions of PMP 22 gene and mutations in the following genes: early growth response 2, connexin 32, mitofusin, myelin protein zero and transient receptor potential cation channel subfamily V member 4. Molecular analysis (through PCR amplification and direct sequencing) of the *PMP22* gene was performed. All coding exons and flanking intronic sequences of the *PMP22* gene were analysed, except the 5′ UTR and 3′ UTR, reference sequences (primers) for PMP22 messenger: NM_000304, for peripheral myelin protein 22: NP_000295. We found a nucleotidic substitution c.178 + 2 T > C on intron 3, involving the splicing donor site, not reported in the following databases: dbSNP, EVS, ExAC. The variant was considered likely pathogenic. It is predicted to result in alteration of the wild type donor site, most probably affecting splicing by breaking the wild type donor site (source: human splicing finder, prediction algorithms: HSF Matrices, MaxEnt). No other variants were identified. A segregation analysis was undertaken on both parents, retrieving negative results in the mother and demonstrating the same mutation (nucleotidic substitution c.178 + 2 T > C on intron 3) in the patient’s father.

The last nerve conduction study, performed 6 months later, confirmed previous results, but the right motor radial nerve examination showed normal values also when compared with left radial nerve values. The numbness disappeared after 1 week from the onset and no further symptoms occurred. The last neurological examination (performed 1 year later) was normal: in particular, no hypoesthesia or hyposthenia in the right hand were noticed.

The patient’s father is a 43 years old man reporting recurrent episodes of prolonged numbness in the ulnar and radial nerve distribution and exercise-induced myalgia for which he never sought medical attention. No weakness episodes were reported. His neurological examination showed lower limb areflexia, distal hypotrophy in his lower limb, mild bilateral pes cavus and mild hypostenia of peroneal ant tibial muscles.

Electrophysiological examination showed symmetrical demyelinating sensory and motor chronic polyneuropathy, predominant in the lower limb and more severe in the sensory nerves. Partial conduction block was also present in both tibial posterior nerves, with no temporal dispersion.

## Discussion and conclusions

We described a family with an hereditary neuropathy with liability to pressure palsy presenting in a 4-years-old girl with acute onset of focal numbness and weakness in the right hand, secondary to a new mutation of the *PMP22* gene, inherited from her affected father.

From a clinical point of view the transient sensory symptoms associated with focal motor mononeuropathy is very atypical at onset, especially in the paediatric age. Usually, age at onset of first symptoms is in the second or third decade. The early and uncommon presentation here reported expands the clinical scenario of HNPP disease and draws attention to the association of mild symptoms in an otherwise healthy child without a clinical history suggestive for inherited neuropathy. Previous, genetically-confirmed, early-onset cases have been reported in the literature, ranging from the neonatal period (presenting with foot drop [11] or Erb brachial plexus palsy [12]) to a 2-years-old boy with acute severe weakness of one hand, no known family history, but with revealing neurophysiologic findings (neuropathy at entrapment site associated with a nerve conduction velocity slowing at other sites) [13]. The phenotypical heterogeneity in the paediatric age is very wide, comprising profound hypotonia and bilateral, self-limited foot drop at birth, followed by generalized hypotonia and mild weakness, resulting in gross motor delay in infancy [14]. Variable clinical presentations have been described in a paediatric cohort (4–18 years of age), from mononeuropathies (radial, fibular or musculocutaneous) to brachial plexopathy, with recurrent episodes in 57% of patients [15].

A clinically acute onset with transient sensory and motor radial nerves involvement was not reported in paediatric patients. In fact, a motor neuropathy or recurrent painless sensory episodes are the most frequent symptoms described in the great majority of patients, albeit in adults. Other chronic symptoms such as cramps and exercise-induced myalgia reported by the father are more common in adult patients [7] as well as cranial nerves involvement or chronic motor deficits. On clinical examination, the presence of mild atrophy and areflexia as noticed in the father have been reported, although uncommon [8].

The electrophysiological findings of the child suggestive for a mixed (predominantly demyelinating) polyneuropathy with sensory-motor involvement are in keeping with a diagnosis of HNPP. The typical patterns found in the paediatric age have been recently described: multifocal demyelination at the entrapment site, generalized demyelinating sensory-motor polyneuropathy and a combination of these two patterns [6], although different patterns more suggestive for an axonal sensory neuropathy and a mononeuropathy with conduction block have also been reported [16]. The peculiarity of the electrophysiological findings in this child was the overlapping of a transient radial nerve palsy in association with chronic polyneuropathy. This pattern is singular and highlights the neurophysiological variability of this disorder in paediatric patients. Furthermore, in contrast with previous literature, considering the presence of a carpal tunnel syndrome as a diagnostic criterium in adults [8] and seeming to confirm its diagnostic relevance in children (with universal electrophysiological positivity in tested patients) [6], we found normal median nerve CMAP values in our patient and in her father.

Finally, *PMP22* point mutations can lead to either a CMT or a HNPP phenotype or even to overlapping phenotypes. Several types of *PMP22* point mutations are described (missense, nonsense, frameshift and splice-site mutations) in association with an HNPP phenotype. These cases often result in either premature or delayed stop codons, determining a null allele [17], in line with gene dosage as the pathogenic mechanism in HNPP as well as in CMT1A [18]. However, the phenotype in HNPP patients carrying point mutations is often atypical when compared to patients carrying deletions [19], therefore some authors argue that HNPP due to *PMP22* point mutations should be seen as a separate entity from HNPP due to *PMP22* deletions [18]. For example, the frameshift variant p.Arg95GlyfsTer128, although associated with a typical HNPP phenotype, also more likely gives rise to associated neuropathic symptoms and signs mimicking CMT1 [19]. A similar phenotype has been described in association with other single-nucleotide variants [20–25]. Additionally, a severe, atypical “HNPP-like” phenotype was described in association with the p.Thr118Met pathogenic variant [26], previously reported as either a cause of an autosomal recessive CMT1 [27] or as a benign polymorphism [28]. Severely affected children, compound heterozygous for two different *PMP22* deletions and showing a CMT phenotype have also been described [29, 30]. In our case the normal neurological examination of the child and the mild progression of clinical signs in the father fulfill the clinical criteria for HNPP disorder. Although nucleotidic substitutions involving either splicing acceptor or donor sites [31] have already been described, to the best of our knowledge, this specific point mutation has not been previously reported, thus adding to current knowledge.

In conclusion, to the best of our knowledge, this specific point mutation in *PMP22* gene has not been previously reported. In the presence of a child with a sensory and motor transient episode, HNPP disorder due to *PMP22* mutations should be suspected. Furthermore, the clinical and electrophysiological studies should be extended to all the family members also in the absence of previous episodes suggestive for an hereditary neuropathy with liability to pressure palsy.

## Abbreviations

CMT: Charcot-Marie Tooth
dbSNP: The database of short genetic variation
EVS: Exome variant server
ExAC: Exome aggregation consortium
HNPP: Hereditary neuropathy with liability to pressure palsy
Mb: Mega-bases
PMP22: Peripheral myelin protein 22 gene
